# Dynamic transition of chemolithotrophic sulfur-oxidizing bacteria in response to amendment with nitrate in deposited marine sediments

**DOI:** 10.3389/fmicb.2015.00426

**Published:** 2015-05-18

**Authors:** Tomo Aoyagi, Makoto Kimura, Namiha Yamada, Ronald R. Navarro, Hideomi Itoh, Atsushi Ogata, Akiyoshi Sakoda, Yoko Katayama, Mitsuru Takasaki, Tomoyuki Hori

**Affiliations:** ^1^Environmental Management Research Institute, National Institute of Advanced Industrial Science and TechnologyTsukuba, Japan; ^2^Bioproduction Research Institute, National Institute of Advanced Industrial Science and TechnologySapporo, Japan; ^3^Institute of Industrial Science, The University of TokyoTokyo, Japan; ^4^Graduate School of Agriculture, Tokyo University of Agriculture and TechnologyTokyo, Japan; ^5^Department of Food and Environmental Sciences, Faculty of Science and Engineering, Ishinomaki Senshu UniversityIshinomaki, Japan

**Keywords:** marine sediment, sulfur-oxidizing bacteria, environmental stimuli, high-throughput sequencing, the Great East Japan Earthquake in 2011

## Abstract

Although environmental stimuli are known to affect the structure and function of microbial communities, their impact on the metabolic network of microorganisms has not been well investigated. Here, geochemical analyses, high-throughput sequencing of 16S rRNA genes and transcripts, and isolation of potentially relevant bacteria were carried out to elucidate the anaerobic respiration processes stimulated by nitrate (20 mM) amendment of marine sediments. Marine sediments deposited by the Great East Japan Earthquake in 2011 were incubated anaerobically in the dark at 25∘C for 5 days. Nitrate in slurry water decreased gradually for 2 days, then more rapidly until its complete depletion at day 5; production of N_2_O followed the same pattern. From day 2 to 5, the sulfate concentration significantly increased and the sulfur content in solid-phase sediments significantly decreased. These results indicated that denitrification and sulfur oxidation occurred simultaneously. Illumina sequencing revealed the proliferation of known sulfur oxidizers, i.e., *Sulfurimonas* sp. and Chromatiales bacteria, which accounted for approximately 43.5% and 14.8% of the total population at day 5, respectively. These oxidizers also expressed 16S rRNA to a considerable extent, whereas the other microorganisms, e.g., iron(III) reducers and methanogens, became metabolically active at the end of the incubation. Extinction dilution culture in a basal-salts medium supplemented with sulfur compounds and nitrate successfully isolated the predominant sulfur oxidizers: *Sulfurimonas* sp. strain HDS01 and *Thioalkalispira* sp. strain HDS22. Their 16S rRNA genes showed 95.2–96.7% sequence similarity to the closest cultured relatives and they grew chemolithotrophically on nitrate and sulfur. Novel sulfur-oxidizing bacteria were thus directly involved in carbon fixation under nitrate-reducing conditions, activating anaerobic respiration processes and the reorganization of microbial communities in the deposited marine sediments.

## Introduction

Environmental stimuli affect the structure and function of microbial communities in natural environments. Numerous investigations have employed artificial stimulations by temperature, pH, NaCl, and electron donors and acceptors to clarify which microorganisms play important roles in the development and functioning of microbial communities (Hori et al., 2006; Kotsyurbenko et al., 2007; Lydmark et al., 2007; Ontiveros-Valencia et al., 2012). For instance, in terrestrial ecosystems, the supplementation with inorganic electron acceptors, e.g., nitrate, iron(III), and sulfate, has been shown to change the metabolic pathway and/or strengthen the degradation capability of indigenous microbial communities (Lueders and Friedrich, 2002; Labrenz et al., 2005; Hori et al., 2010), which in turn provides an opportunity to study the relationship among biogeochemical cycles of nitrogen, iron, sulfur, and carbon. However, the microbial community adaptation and development in response to environmental stimuli remain to be delineated because of the limited information concerning microbial population dynamics, rather than the dynamics of a particular taxon or a specific trophic group.

Marine sediment is commonly found on the sea floor below fish farms and in closed water areas throughout the world (Pearson and Rosenberg, 1978; Kemp et al., 1990; Armenteros et al., 2010; Kunihiro et al., 2011). A large quantity of marine sediment was deposited by the tsunami originating from the Great East Japan Earthquake in 2011, and the study of this material has shed light on the vast accumulation of marine sediments in not only closed sea areas but also coastal marine regions facing the open ocean (Inui et al., 2012). The geochemical cycles of elements in marine sediments have attracted much attention because of their possible association with coastal biotic activities. In a recent study, we reported that only limited degradation of organic matter occurred in association with the sulfate reduction in deposited marine sediments, even though the 16S rRNA transcripts detected were mainly from sulfate reducers (Hori et al., 2014). In the same report, we found that ferric iron amendment had little effect on the structure and activity of the sediment microorganisms. Marine sediments have been accumulating in the past few decades, possibly due to their resistance to the degradation abilities of sulfate and iron reducers. For this reason, biostimulation with energetically more favorable electron acceptors such as nitrate is considered more effective in facilitating degradation processes in the marine sediments. This is particularly relevant because nitrate pollution from farm run-off and/or other sources may currently be contributing to the nitrate amendment of marine sediments. However, the precise effects of such biostimulation and the responsible microorganisms are largely unknown.

The advent of high-throughput DNA sequencing technologies has revolutionized approaches to the study of microbial community structure and function (Caporaso et al., 2011; Miller et al., 2013). One of these technologies, the Illumina platform for 16S rRNA amplicon sequencing, has made it possible to comprehensively screen microbial communities at high resolution and high sensitivity (Caporaso et al., 2012). In addition, the comparative analysis of 16S rRNA genes and transcripts has enabled the fine-scale identification of metabolically active microorganisms. The resulting phylogenetic information is useful for the isolation of key microorganisms, which is one of the exclusive means of accessing their ecophysiological roles.

The objective of this study was to amend deposited marine sediments with an energetic electron acceptor, nitrate, and investigate the impact of this amendment on the structure and activity of microbial communities by using a combination of geochemical analyses, high-throughput Illumina sequencing of 16S rRNA genes and transcripts, and isolation.

## Materials and Methods

### Anaerobic Incubation of the Deposited Marine Sediments

Marine sediments deposited by the tsunami that originated from the Great East Japan Earthquake in 2011 were collected on September 2011. The sampling site was located on the coast of Higashi-matsushima, Miyagi, Japan (38°25′N, 141°14′E). The samples were stored at 4°C prior to experimental use. Sediment slurry was prepared by mixing the deposited sediment with artificial seawater (Daigo; Nihon Pharmaceutical) at a ratio of 1:4 (v/v). Aliquots (20 ml) of the homogenized slurry were transferred anaerobically into 50-ml serum vials, which were then sealed with butyl rubber septa. The samples were pre-incubated in the dark at 25°C for more than 30 days in order to activate the sediment microorganisms. After pre-incubation, the headspace of each sample vial was flushed with N_2_. Three sediments were produced: (i) a non-autoclaved sediment amended with nitrate to a final concentration of 20 mM, (ii) a non-autoclaved sediment with addition of sterilized water in place of nitrate (control sediment), and (iii) an autoclaved sediment amended with nitrate to a final concentration of 20 mM. Sterilization of the sediment microorganisms was performed three times at 121°C for 1 h. All treatments were run in triplicate with static incubation for 5 days at 25°C in the dark.

### Geochemical Analyses for Gas, Liquid, and Solid Phases of the Sediment Incubation

Samples of the headspace gas, slurry water and solid-phase sediment were taken at day 0, 2, and 5 from each vial of each of the triplicate replications of the two treatments (i.e., the nitrate and non-amended control treatments) performed in triplicate. Total CO_2_, N_2_O, and CH_4_ in the headspace gas were analyzed by a gas chromatograph (GC-14B; Shimadzu) equipped with a packed column (ShinCarbon ST; Shinwa). Concentrations of NO_3_^-^, NO_2_^-^, NH_4_^+^, PO_4_^2-^, and SiO_2_ in the slurry water were determined by the colorimetric method (Hansen and Koroleff, 2007) with an auto-analyzer (QuA Atro 2-HR; BLTEC). This colorimetric analysis was also conducted on the nitrate-amended sterilized sediments after a 5-day incubation. The sulfate concentration in the slurry water was determined by using an ion chromatograph (DX-500; Dionex) equipped with an IonPac AS11 column (Dionex) and an ED40 electrochemical detector (Dionex). The total organic carbon (TOC) concentration from the slurry water was determined by the non-purgeable organic carbon method with a TOC analyzer (TOC-L; Shimadzu). The concentration of volatile fatty acids (VFAs) from the slurry water was measured by a high-pressure liquid chromatograph (Alliance e26951; Waters) equipped with an RSpak KC-811 column (Shodex) and a photodiode array (2998; Waters). Ferrous iron (Fe^2+^) and total iron in the slurry were determined using 6N HCl extraction and the ferrozine method as described previously (Braunschweig et al., 2012). The content ratio of elements (i.e., carbon, hydrogen, nitrogen, and sulfur) in the solid-phase sediment was determined by using a CHNS analyzer (FLASH 2000 Organic Elemental analyzer; Thermo Scientific). The thermogravimetry (TG) and differential thermal analysis (DTA) curves of the solid-phase sediment were determined by using a simultaneous differential thermo gravimetric analyzer (DTG-60; Shimadzu). The sediment slurry samples were stored at –80°C for subsequent molecular analyses.

### Nucleic Acid Extraction and Amplification of 16S rRNA Genes and Transcripts

Nucleic acids were extracted from each sample from one replication of the nitrate and control treatments at day 0, 2, and 5 using a direct lysis protocol involving bead beating (Noll et al., 2005). Total DNA and RNA were prepared by digestion with RNase (Type II-A; Sigma-Aldrich) and DNase (RQ1; Promega), respectively. The PCR was performed with a high-fidelity DNA polymerase (Q5; New England Biolabs). Reverse transcription PCR (RT-PCR) was carried out using a one-step RT-PCR system (Access Quick; Promega). The universal primer set 515F/806R was modified to contain an Illumina adaptor region, and the reverse primer was encoded with 6-bp barcodes (Caporaso et al., 2012). The thermal profile of PCR was as follows: an initial denaturation at 98°C for 90 s; followed by 25 cycles of denaturation at 98°C for 10 s, annealing at 54°C for 30 s, and extension at 72°C for 30 s; and a final extension step at 72°C for 2 min. For RT-PCR, RT was performed at 48°C for 45 min, and the thermal profile of the subsequent PCR consisted of a denaturation at 94°C for 3 min; followed by 30 cycles of 30 s at 94°C, 45 s at 52°C, and 90 s at 72°C; with a final extension of 5 min at 72°C. The absence of the DNA contamination was confirmed by RT-PCR without reverse transcriptase.

### Illumina Sequencing and Data Processing

The amplicons were purified first with an AMPure XP Kit (Beckman Coulter) and then a second time with a QIAquick gel extraction kit (QIAGEN). The barcode-encoded DNA library and an initial control (PhiX; Illumina) were subjected to paired-end sequencing with a 300-cycle MiSeq Reagent kit (Illumina) on a MiSeq sequencer (Illumina). The PhiX, low-quality (Q < 30), and chimeric sequences were removed and the paired-end sequences were assembled as described previously (Itoh et al., 2014). The sequences in each library were characterized phylogenetically using the QIIME software package version 1.7.0 (Caporaso et al., 2010). The operational taxonomic units (OTUs) were grouped using a 97% sequence identity cut-off. Representative sequences for each OTU were assigned with the BLAST program in the DDBJ nucleotide sequence database. Using the program QIIME (Caporaso et al., 2010), α-diversity indices (i.e., Chao1, Shannon, and Simpson reciprocal) and the weighted UniFrac distances for principal coordinate analysis (PCoA) were calculated.

### Isolation and Phylogenetic Analysis of the Dominant Sulfur Oxidizers

For isolation and cultivation of the dominant sulfur-oxidizing bacteria from the deposited marine sediments, the incubated slurry was transferred to a slightly modified Widdel medium (pH 7.0; Kanno et al., 2013) containing NaCl, MgCl_2_⋅6H_2_O, KH_2_PO_4_, and NH_4_Cl at concentrations of 20.5, 3, 0.136, and 0.005 g/l, respectively. In this protocol, 2 ml of the incubated slurry and 18 ml of basal medium were first distributed into 50 ml glass serum vials with butyl rubber septa. Then the solutions were further amended with nitrate (20 mM) as an electron acceptor and elemental sulfur (20 mM) or thiosulfate (20 mM) as an electron donor. After flushing the vial headspace with N_2_/CO_2_ (80: 20, v/v), enrichment was performed at 25°C in the dark. After 1 week, 5% of the enrichment cultures were transferred to fresh media, and the subculturing process was carried out four times. Thereafter, extinction dilution culture was conducted for the isolation of sulfur oxidizers. The purification of isolates was checked by microscopic observation and Illumina sequencing of 16S rRNA genes.

For the phylogenetic analysis of isolates, nucleic acids were extracted from the pure cultures as described above. Nearly the full length of the 16S rRNA gene sequences was amplified with PCR using a Q5 DNA polymerase (New England Biolabs) and the primer set B27f/B1525r (Lane, 1991). The thermal conditions of PCR were as described above, except that an annealing temperature of 52°C and an extension time of 1 min were employed. The PCR products were purified and ligated into the plasmid vector pGEM-T Easy (Promega), and the ligation mixture was used to transform *Escherichia coli* JM109 supercompetent cells (Nippon Gene) according to the manufacturer’s instructions. The plasmid DNA was sequenced using a BigDye Terminator v3.1 Cycle Sequencing kit (Applied Biosystems) and a 3730xl DNA Analyzer (Applied Biosystems). A phylogenetic tree was constructed by the neighbor-joining and maximum likelihood methods using the program MEGA, version 5.2 (Tamura et al., 2011). The robustness of the tree topology was assessed by the bootstrap value based on 1,000 replications. For physiological characterization, the sulfate concentrations during the incubation with sulfur compounds (i.e., elemental sulfur and thiosulfate) and nitrate were determined to assess the chemolithotrophic growth of the isolates.

### Nucleotide Sequence Accession Numbers

The nucleotide sequence data obtained from Illumina sequencing analyses based on the 16S rRNA genes and transcripts have been deposited in the MG–RAST database^1^ under the project title “Dynamic transition of chemolithotrophic sulfur oxidizers in deposited marine sediments in 2015” with the ID numbers 4620652.3–4620661.3 (10 libraries), and those obtained from the phylogenetic analysis of isolates have been deposited in the DDBJ nucleotide sequence database^2^ under accession numbers LC029406 to LC029408 (three isolates).

## Results

### Biogeochemical Activities in the Nitrate-Amended Marine Sediments

**FIGURE 1:**
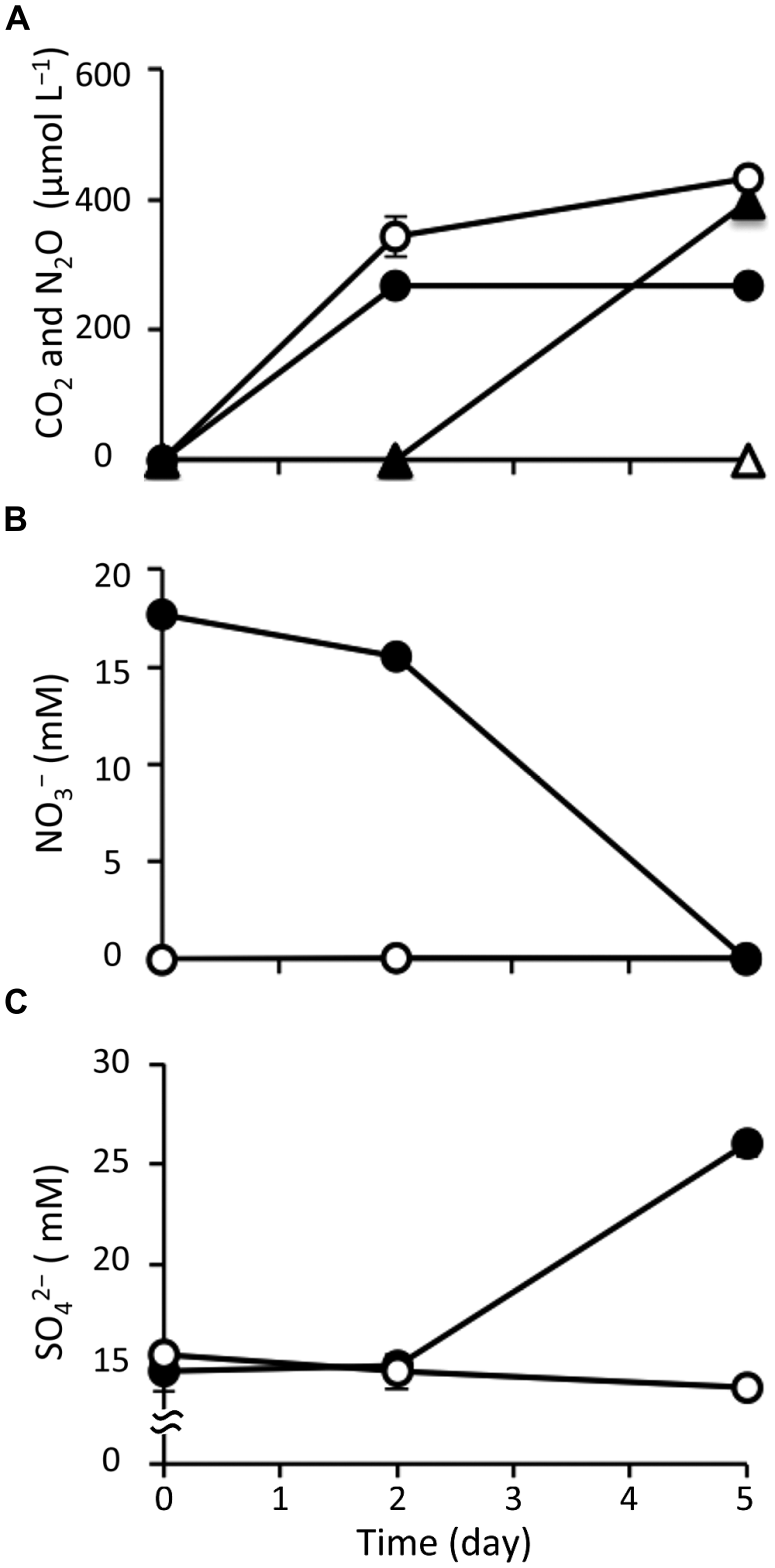
**Changes in geochemical parameters during anoxic incubation of nitrate-amended marine sediments (closed symbols) and the control (opened symbols).** The panels show the concentrations of CO_2_ (circle) and N_2_O (triangle) **(A)**, nitrate **(B)**, and sulfate **(C)**. The error bars indicate the standard deviations of three replications.

After amending with nitrate at a concentration of 20 mM, marine sediment slurries were anaerobically incubated for 5 days; this was designated the nitrate treatment. In parallel, the incubation of non-amended slurries was conducted as a control. Geochemical analysis showed significant differences in the biological activity under the different incubation conditions. First, based on the gaseous phase analysis of the samples (**Figure 1A**), N_2_O was produced from day 2 in the nitrate-amended sediment but it was not detected in the control. Under both conditions, CO_2_ was generated from the start of the incubations. In both cases the CO_2_ concentrations increased to 258–378 μM at day 2, suggesting that the CO_2_ production was caused by degassing and establishment of a CO_2_-bicarbonate equilibrium. After 2 days, the CO_2_ concentration stabilized at 255–285 μM under the nitrate treatment, while it increased to 434 μM at day 5 in the control. CH_4_ was not detected under either condition throughout the incubation (data not shown).

With respect to the analysis of slurry water in the nitrate-amended samples, the nitrate concentration decreased to 15.5 mM at day 2 and was completely depleted at the end of the incubation (**Figure 1B**). Nitrite was not detected during the 5-day incubation (data not shown). The ammonium concentration increased slightly to 1.25 mM at day 2, and then decreased to 0.78 mM at day 5 (Supplementary Figure S1A). Sulfate concentrations were maintained at 14.6–15.5 mM during the first 2 days, but they increased remarkably to 26.0 mM at day 5 (**Figure 1C**). This suggests that sulfur components originally present in the sediment were gradually being oxidized to sulfate. TOC was kept at a constant level of 22.6 mg l^-1^ during the incubation (Supplementary Figure S1B). Since VFAs were absent in the slurry water, they were considered not to have contributed to the TOC content. The concentrations of silicate and phosphate, which are known nutrients for microbial growth, decreased throughout the incubation period (Supplementary Figures S1C,D). It was interesting to note that these geochemical parameters did not change in the control (**Figure 1** and Supplementary Figure S1). In addition, decreases in nitrate, silicate, and phosphate were also not observed in the nitrate-amended sterilized sediments (data not shown). These findings indicate that the apparent denitrification and sulfur oxidation under the nitrate treatment were microbially mediated processes.

**FIGURE 2:**
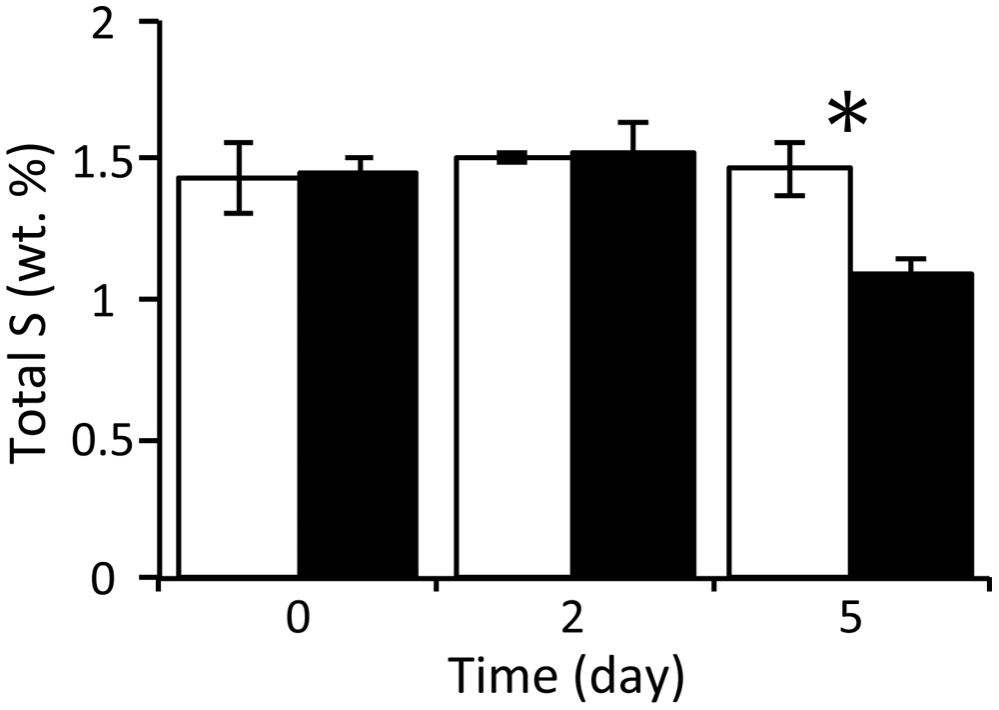
**Changes in the content ratios of sulfur elements in the solid-phase sediments during anoxic incubation of nitrate-amended marine sediments (black bar) and the control (white bar).** The error bars indicate the standard deviations of three replications. Asterisks indicate significant differences in content ratios between the two treatments (^∗^*p* < 0.05).

With respect to the analysis of solid-phase sediments, the content ratio of the sulfur moiety decreased to 1.1 wt% at day 5 under the nitrate treatment, and this value was significantly lower than that in the control (*P* < 0.05; **Figure 2**). Combined with the results from the slurry water analyses, this strongly suggests that the reduced sulfur components in the nitrate-amended sediments were oxidized to sulfate which was then released to the liquid phase. No significant differences in the content ratios of the other elements (i.e., carbon, hydrogen, and nitrogen) were observed between the nitrate treatment and the control (Supplementary Figure S2). The TG/DTA curves also showed that there were no significant differences in these ratios from the beginning (Supplementary Figure S3A) until the end of the incubation under either set of conditions (Supplementary Figures S3B,C), which suggests that the major components of the sediments were little changed by nitrate amendment.

### Changes in Microbial Communities Based on 16S rRNA Genes and Transcripts

**FIGURE 3:**
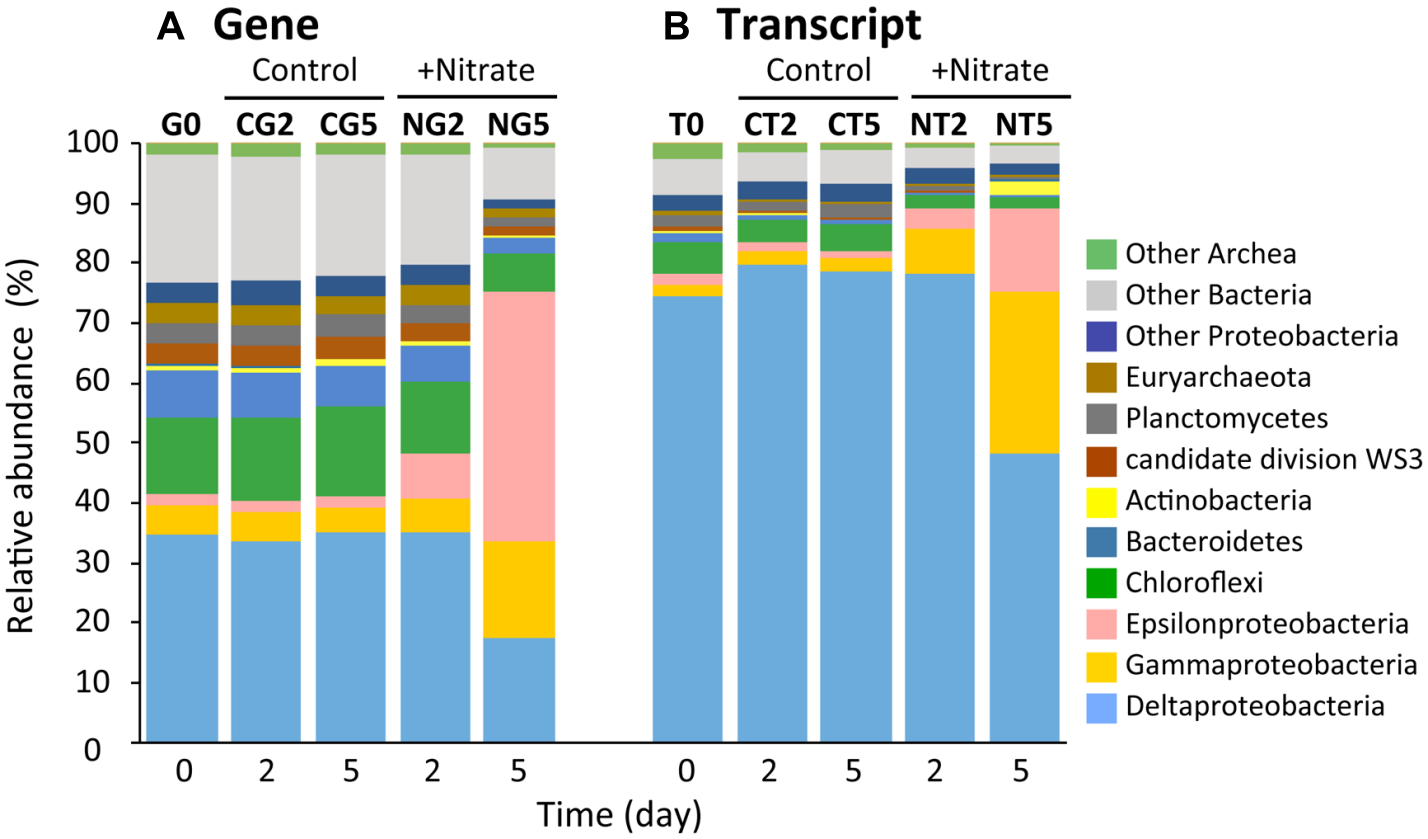
**Dynamic transition of microbial communities during anoxic incubation of marine sediments as determined by Illumina sequencing of 16S rRNA genes **(A)** and transcripts (B).** Relative abundances of 16S rRNA genes (‘G’) and transcripts (‘T’) defined phylogenetically are represented by the colors shown at the right side of the graph. The sequence libraries were obtained from the incubation at day 0 (designated the G0 and T0 libraries) and at days 2 and 5 in the nitrate-amended sediment (‘N’; designated the NG2, NG5, NT2, and NT5 libraries) and in the control (‘C’; designated the CG2, CG5, CT2, and CT5 libraries). The sequence library is indicated at the top of each bar.

Illumina sequencing on the basis of the 16S rRNA genes and transcripts demonstrated the dynamic transition of whole and metabolically active microbial communities in the nitrate-amended sediments (**Figure 3** and Supplementary Table S1). A total of 10 Illumina sequence libraries were constructed from 16S rRNA genes (‘G’) and transcripts (‘T’) at day 2 and 5 for both the nitrate (‘N’) treatment and the control (‘C’). For instance, the library built from 16S rRNA genes at day 2 of the nitrate treatment was designated as the NG2 library. The NG5, NT2, NT5, CG2, CG5, CT2, and CT5 libraries were defined in the same manner. The libraries from 16S rRNA genes and transcripts at day 0 were presented as a consensus between the two conditions, and were designated as the G0 and T0 libraries, respectively. A total of 280,651 sequences (i.e., a mean of 28,065 sequences per library) were characterized phylogenetically. The numbers of sequences in all the libraries are shown in Supplementary Table S1.

Alpha-diversity indices (i.e., Chao1, Shannon, and Simpson reciprocal) of the Illumina libraries were determined on the basis of equal numbers of sequences (*n* = 7,374; Supplementary Table S1). The Chao1 values in the NG2, NG5, NT2, and NT5 libraries were significantly lower than those in the CG2, CG5, CT2, and CT5 libraries, respectively, which indicates that the richness of microbial communities decreased in response to nitrate amendment. Further, the values of the indices Shannon and Simpson reciprocal were in the order G0 > NG2 > NG5 libraries, while those for the G0, CG2, and CG5 libraries were at similar levels. A decreasing trend was also observed in the T0, NT2, and NT5 libraries but not in the T0, CT2, and CT5 libraries. These facts show that the even distribution of microbial communities significantly decreased with time following the nitrate treatment. The decrease in the evenness of the distribution was more obvious in the whole microbial populations (i.e., at the gene level) than in metabolically active microorganisms (at the transcript level). Taken together, these results showed that the microbial diversity was remarkably lowered by nitrate amendment and thus indicated that particular species dominated the microbial communities. In order to compare the community structures in all the libraries, a PCoA plot was generated from the weighted UniFrac distance analysis using equal numbers of sequences (*n* = 9,336; Supplementary Figure S4). In the nitrate treatment, distance was maintained between all the libraries on the plot, while in the control treatment, the libraries were in close proximity. This suggests that the microbial communities shifted progressively with time in the nitrate-amended sediments. A considerably greater degree of change in the community was observed from days 2–5 compared to days 0–2. In addition, the libraries of 16S rRNA genes were located far from those of the transcripts, implying that only selected members of the microbial communities expressed 16S rRNA.

**FIGURE 4:**
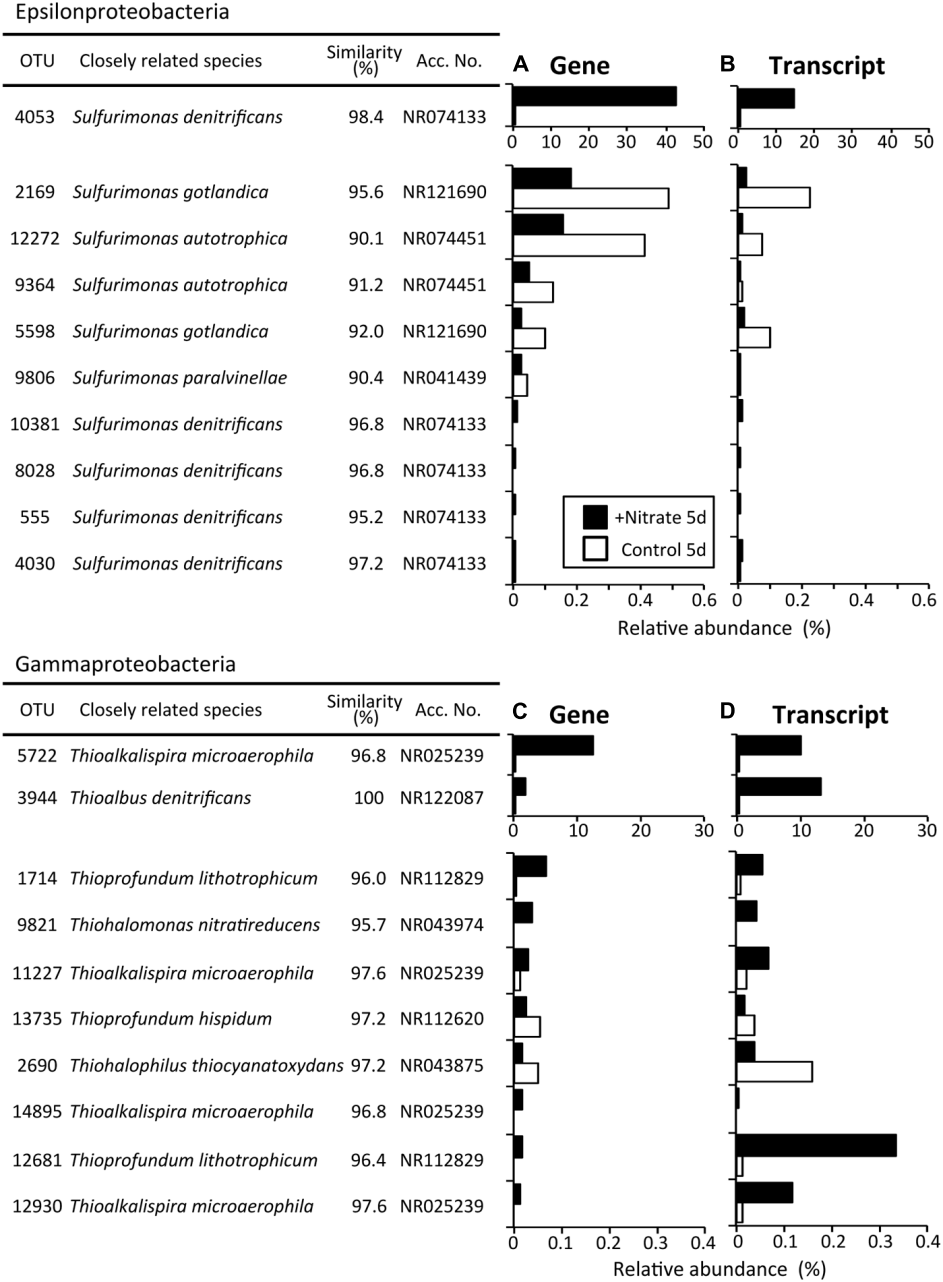
**The most abundant operational taxonomic units (OTUs) of the classes Epsilonproteobacteria **(A)** and Gammaproteobacteria **(C)** after the 5-day anoxic incubation of nitrate-amended marine sediments (black bar) and the control (white bar).** OTUs are listed in descending order of relative abundance in the NG5 library **(A,C)**, and the transcript levels in the NT5 library are shown **(B,D)**. White bars indicate their relative abundances in the CG5 and CT5 libraries.

Phylogenetic analyses of the Illumina sequence libraries revealed the drastic changes in microbial community structures following the nitrate treatment (**Figure 3**). Notably, the Epsilon- and Gamma-proteobacteria classes increased drastically from 2% and 5% of the whole microbial population at day 0 to 44% and 17% at day 5, respectively (G0 and NG5 libraries in **Figure 3A**). Significant increases in these classes were also found in the metabolically active microbial communities (T0 and NT5 libraries in **Figure 3B**). The most abundant OTUs of these classes in the NG5 library are shown in **Figure 4**. Within the class Epsilonproteobacteria, OTU 4053 became the most predominant, accounting for 42.7% and 14.7% of the total NG5 and NT5 libraries, respectively (**Figures 4A,B**). This OTU was phylogenetically related to *Sulfurimonas denitrificans* (NR074133; 98.4% sequence similarity). Concerning the Gammaproteobacteria, the Chromatiales OTUs 5722 and 3944 accounted for 12.5% and 1.9%, respectively, of the total in the NG5 library (**Figure 4C**). The closely related species of these OTUs were *Thioalkalispira microaerophila* (NR025239; 96.8% sequence identity) and *Thioalbus denitrificans* (NR122087; 100% sequence identity), respectively. On the other hand, the epsilon- and gamma-proteobacterial OTUs are listed in descending order of relative abundance in the NT5 library (Supplementary Figure S5). Only small differences in the abundant epsilonproteobacterial OTUs were observed between the NT5 and NG5 libraries (**Figure 4A** and Supplementary Figure S5B), indicating that 16S rRNA expression was mostly originated from the major members, more specifically the OTU 4053, within the Epsilonproteobacteria. Meanwhile, the abundant gamma-proteobacterial OTUs in the NT5 library were remarkably distinct from those in the NG5 library (**Figure 4C** and Supplementary Figure S5D). The metabolic activity of the community members appeared not to be linked with the population size, and a variety of minor microorganisms within the Chromatiales highly expressed 16S rRNA. Consequently, the high-resolution dynamics of microbial communities on the basis of the 16S rRNA genes and transcripts revealed that the specific members within the genus *Sulfurimonas* and the order Chromatiales, hitherto known as sulfur-oxidizing bacteria (Friedrich et al., 2001; Nakagawa et al., 2005; Campbell et al., 2006; Nakagawa and Takai, 2008), drastically proliferated and/or were metabolically activated in the nitrate-amended sediments. Moreover, additional quantification assays, e.g., quantitative PCR, of 16S rRNA genes and transcripts are effective for verifying the relationship between the population sizes and metabolic activities of the sulfur-oxidizing bacteria.

Some OTUs outside the Epsilon- and Gamma-proteobacteria exhibited higher levels of 16S rRNA expression in the NT5 library than in the CT5 library. The OTUs with more than five-fold increases in relative abundance are shown in **Table 1**. The metabolic activation of anaerobically respiring microorganisms other than sulfur oxidizers was observed at the end of the incubation. In particular, the 16S rRNA expression level of the fermentative and heterotrophically nitrate-reducing *Cellulomonas* sp. (OTU: 566) in the NT5 library was 430-fold higher than that in the CT5 library. The iron(III)-reducing bacteria such as the *Geobacter* spp. (OTUs: 8007, 425, and 3917), *Pelobacter* sp. (OTU: 2865), and *Geothrix* sp. (OTU: 2401) showed 13–94-fold increases in relative abundance. In addition, the 16S rRNA expressions from the syntrophically VFA-oxidizing Syntrophobacteriaceae bacterium (OTU: 11860) and the aceticlastic methanogen *Methanosaeta* sp. (OTU: 3178) increased to some extent in the NT5 library compared to those in the CT5 library (data not shown).

**Table 1:** Operational taxonomic units (OTUs) highly expressing 16S rRNA after the 5-day anoxic incubation of nitrate-amended marine sediments.

OTU ID	Closely related species	Similarity (%)	Accession number	Phylum/Class	Family	Relative abundance (%)^a^	Increasing ratio (fold)^b^	Putative function^c^
4053	*Sulfurimonas denitrificans*	98.4	NR121690	Epsilonproteobacteria	Helicobacteraceae	14.64	2421.7	Sulfur oxidation
566	*Cellulomonas oligotrophica*	98.8	KF817659	Actinobacteria	Cellulomonadaceae	2.60	430.2	Fermentation, nitrate reduction
13536	*Thioalkalispira microaerophila*	97.6	NR025239	Gammaproteobacteria	Thioalkalispiraceae	0.82	269.8	Sulfur oxidation
11183	*Thioalbus denitrificans*	96.8	NR122087	Gammaproteobacteria	Ectothiorhodospiraceae	0.43	143.1	Sulfur oxidation
8007	*Geobacter bremensis*	100	KF800712	Deltaproteobacteria	Geobacteraceae	6.78	93.5	Iron(III) reduction
3944	*Thioalbus denitrificans*	100	NR122087	Gammaproteobacteria	Ectothiorhodospiraceae	13.37	38.8	Sulfur oxidation
2401	*Geothrix fermentans*	99.2	NR036779	Acidobacteria	Holophagae	0.21	34.7	Iron(III) reduction
14834	*Thioalbus denitrificans*	96.8	NR122087	Gammaproteobacteria	Ectothiorhodospiraceae	0.20	33.7	Sulfur oxidation
7917	*Thioprofundum lithotrophicum*	95.2	NR112829	Gammaproteobacteria	Thioalkalispiraceae	0.09	30.7	Sulfur oxidation
2865	*Pelobacter carbinolicus*	95.2	NR075013	Deltaproteobacteria	Pelobacteraceae	0.09	28.6	Iron(III) reduction, sulfate reduction
425	*Geobacter luticola*	99.6	NR114303	Deltaproteobacteria	Geobacteraceae	6.02	26.5	Iron(III) reduction
20	*Thioprofundum lithotrophicum*	94.5	NR112829	Gammaproteobacteria	Thioalkalispiraceae	0.07	22.5	Sulfur oxidation
12681	*Thioprofundum lithotrophicum*	96.4	NR112829	Gammaproteobacteria	Thioalkalispiraceae	0.33	22.1	Sulfur oxidation
5722	*Thioalkalispira microaerophila*	96.8	NR025239	Gammaproteobacteria	Thioalkalispiraceae	10.17	21.4	Sulfur oxidation
15348	*Thioprofundum lithotrophicum*	95.3	NR112829	Gammaproteobacteria	Thioalkalispiraceae	0.31	17.4	Sulfur oxidation
13687	*Desulfovibrio butyratiphilus*	95.3	NR112679	Deltaproteobacteria	Desulfovibrionales	0.09	15.3	Butyrate oxidation, sulfate reduction
3917	*Geobacter pelophilus*	97.6	NR026077	Deltaproteobacteria	Geobacteraceae	0.12	12.9	Iron(III) reduction
14604	*Desulfocapsa sulfexigens*	93.3	KF952439	Deltaproteobacteria	Desulfobacterales	0.06	10.2	Sulfur disproportionation
12930	*Thioalkalispira microaerophila*	97.6	NR025239	Gammaproteobacteria	Thioalkalispiraceae	0.12	9.7	Sulfur oxidation
17265	*Desulfomonile tiedjei*	98.4	NR074118	Deltaproteobacteria	Syntrophaceae	0.09	7.7	Sulfate reduction
9626	*Desulfobulbus mediterraneus*	94.5	NR025150	Deltaproteobacteria	Desulfobulbaceae	4.05	6.2	Sulfate reduction
1714	*Thioprofundum lithotrophicum*	96.0	NR112829	Gammaproteobacteria	Thioalkalispiraceae	0.06	6.1	Sulfur oxidation

^a^The relative abundance of the 16S rRNA transcripts in the NT5 library. More than 0.05% of the abundance is shown.

^b^The increasing ratio was calculated from the relative abundance in the NT5 library to that in the CT5 library.

^c^The putative function was determined by the reported physiology of the closely related species.

Regarding the composition of microbial communities in the control, the relative abundances in the main phyla or classes were not changed throughout the incubation (G0, CG2, CG5, T0, CT2, and CT5 libraries in **Figure 3**). Deltaproteobacterial sulfate reducers, i.e., Desulfobacteraceae and Desulfobulbaceae bacteria, were dominant. They occupied 11.5% and 7.7% of the CG5 library and 6.3% and 50.3% of the CT5 library, respectively. The dominance of sulfate reducers was consistent with the results from previous studies (Holmer and Kristensen, 1992; Habicht and Canfield, 1997). This suggests that these sulfate-reducing bacteria were originally present in the marine sediments and maintained their predominance throughout the control incubation. Although they expressed most of 16S rRNA under in situ conditions (at day 0) and at days 2 and 5, their contribution to sulfate reduction was small, as indicated by the constant sulfate concentration over time (**Figure 1C**).

### Isolation of the Dominant Sulfur Oxidizers and Their Chemolithotrophic Growth

**FIGURE 5:**
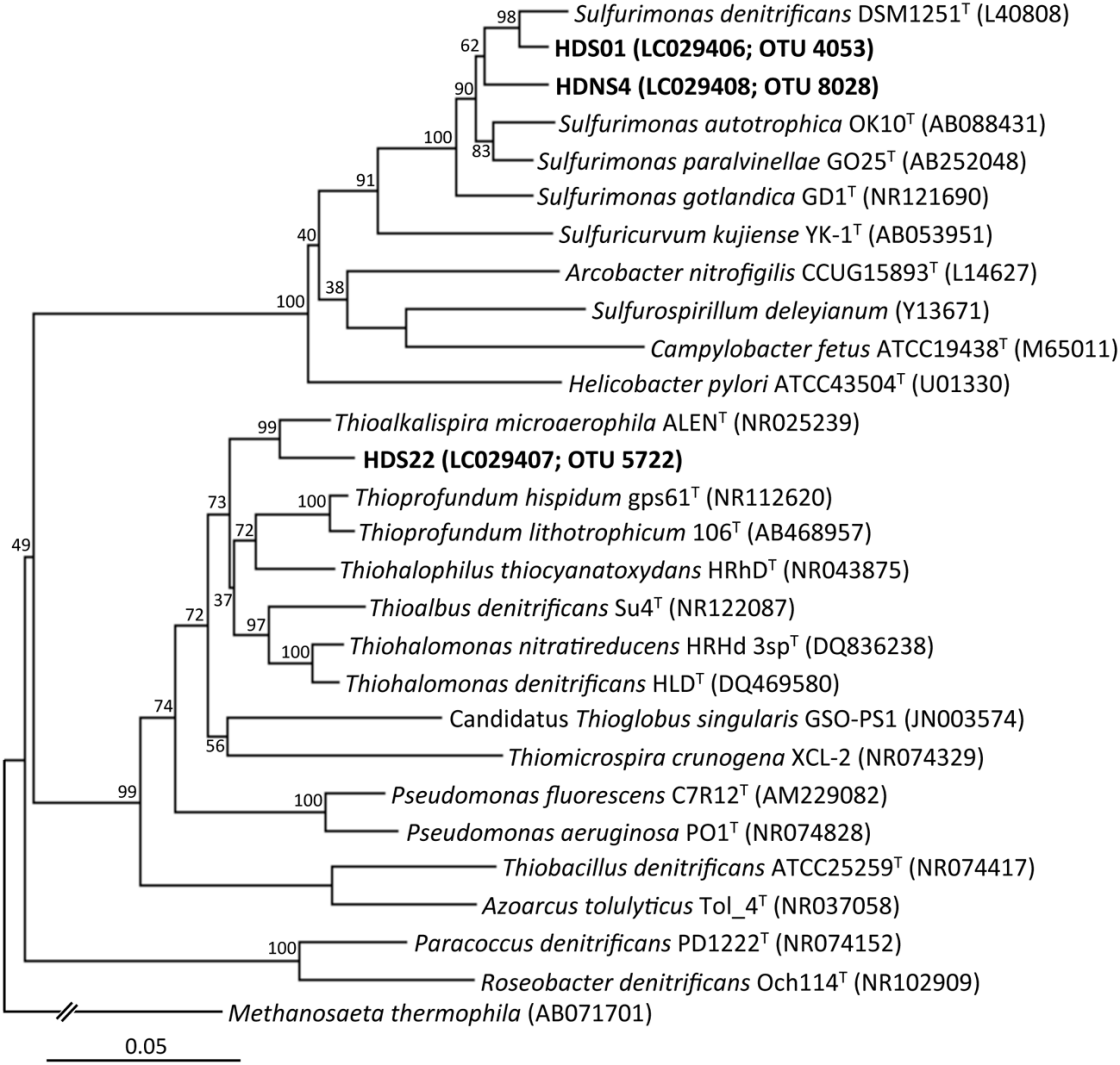
**Phylogenetic tree showing the taxonomic distribution of 16S rRNA genes from isolates (the strains HDS01, HDNS4, and HDS22), and their known cultivated relatives.** The tree was constructed by the neighbor-joining method using the nearly full-length 16S rRNA gene sequences. Bootstrap values were obtained from 1,000 replications. The scale bar represents 5% sequence divergence.

Through extinction dilution culture by using a liquid medium supplemented with nitrate and sulfur compounds, three strains, HDS01, HDS22, and HDSN4, were successfully isolated from the deposited sediments. The phylogenetic analysis based on the nearly full-length 16S rRNA genes indicated that the isolates formed two novel clusters in the phylogenetic tree and the strains HDS01 and HDNS4 were related to *S. denitrificans* (NR074133; 96.7% and 95.8% sequence similarities, respectively), while the strain HDS22 had 95.2% sequence similarity to *Thioalkalispira microaerophila* (NR025239; **Figure 5**). The strains HDS01 and HDS22 corresponded to the OTUs 4053 and 5722, and were identified as the most predominant sulfur oxidizers in the nitrate-amended sediments (**Figures 4A,C**). During the incubation of these two strains (HDS01 and HDS22) with elemental sulfur and thiosulfate as electron donors and nitrate as electron acceptor, the sulfate concentration significantly increased, which indicates that both strains were able to grow chemolithotrophically on these compounds. Because of their low sequence similarities, we consider that these three isolates are novel species in the genera *Sulfurimonas* and *Thioalkalispira*. Further characterizations of these isolates are in progress.

## Discussion

In this study, we investigated microbial community succession stimulated by nitrate amendment of deposited marine sediments. Geochemical analyses were used to monitor the change in the physicochemical parameters of the nitrate-amended sediments (e.g., consumption of nitrate, and production of N_2_O and sulfate). High-throughout Illumina sequencing based on the 16S rRNA genes and transcripts revealed the proliferation and metabolic activation of sulfur-oxidizing bacteria. In addition, isolation of the dominant sulfur oxidizers demonstrated their chemolithotrophic growth on nitrate, which suggests that the biotically fixed carbon was further available for growth of the other anaerobic microorganisms. Actually, at the end of the incubation, Illumina sequencing detected the expression of 16S rRNA from fermentative bacteria, ferric iron reducers, and aceticlastic methanogens. The combination of geochemical analyses, high-throughput sequencing, and isolation was proven to be a powerful approach to comprehensively characterize microbial communities affected by environmental stimuli and their trophic interaction in the marine sediments.

The amendment with nitrate initiated the co-occurrence of sulfur oxidation and denitrification during anoxic incubation of marine sediment slurries (**Figure 1**). Since the concentration of TOC in slurry waters changed little during the incubation (Supplementary Figure S1B), it was suggested that little or no heterotrophic denitrification occurred. There have been reports showing that, in the absence of organic matter as a carbon source, denitrification was coupled with sulfur oxidation in anoxic marine sediments in the Black Sea and the Baltic Sea (Böttcher and Lepland, 2000; Zopfi et al., 2004; Thang et al., 2013). Sulfur compounds that were originally present in the sediments may have served as the electron donor. In this study, the content ratios of sulfur significantly decreased with time in solid-phase sediments amended with nitrate (**Figure 2**). This strongly suggests that certain sulfur compounds such as zerovalent sulfur, thiosulfate, and hydrosulfide ion were oxidized to sulfate in association with denitrification. The oxidation of these compounds and its coupling with denitrification are expressed by the following equations (Cardoso et al., 2006; Zhang et al., 2015):

(1) $${5S}^{0}+{6NO}_{3}^{-}+{2H}_{2}0\to {5SO}_{4}^{2-}+{3N}_{2}+{4H}^{+}\,$$

(2) $${5S}_{2}O_{3}^{2-}+{8NO}_{3}^{-}+H_{2}O\to {10SO}_{4}^{2-}+{4N}_{2}+{2H}^{+}\,$$

(3) $${5HS}^{-}+{8NO}_{3}^{-}+{3H}^{+}\to {5SO}_{4}^{2-}+{4N}_{2}+{4H}_{2}O\,$$

Indeed, throughout the incubation of nitrate-amended sediments, the concentration of nitrate decreased by 17.7 mM, while that of sulfate increased by 11.3 mM (**Figures 1B,C**). The ratio of NO_3_/SO_4_ was 1.57, which is close to the expected stoichiometric value from the equilibrium (3). The accumulation of hydrogen sulfide, the end product of sulfate reduction, has frequently been observed in anoxic marine sediments (Holmer and Kristensen, 1992; Zopfi et al., 2004). This implies that hydrogen sulfide was most likely the electron donor utilized for the redox reaction with denitrification in such environments.

The sulfur oxidizers affiliated with the classes Epsilon- and Gamma-proteobacteria were drastically proliferated in the nitrate-amended sediments, as revealed by Illumina sequencing of 16S rRNA genes and transcripts (**Figure 3**). The predominant OTUs 4053 and 5722 were successfully isolated and were designated as *Sulfurimonas* sp. strain HDS01 and *Thioalkalispira* sp. strain HDS22, respectively. The 16S rRNA genes of these isolates had 95.2–96.7% sequence similarities to those of cultured relatives and were able to grow chemolithotrophically on nitrate and sulfur compounds such as elemental sulfur and thiosulfate. These chemolithotrophic sulfur oxidizers drastically increased during the incubation; the relative abundances of the OTU 4053 (strain HDS01) and the OTU 5722 (strain HDS22) increased from 0.03% and 0.5% at day 0 to 42.7% and 12.5% at day 5, respectively. In addition, the expression levels of the bacterial rRNA were clearly enhanced by nitrate (**Figures 4B,D**). Furthermore, lower CO_2_ concentrations were found in the nitrate-amended sediments than in the non-amended controls, suggesting the occurrence of CO_2_ fixation in the former (**Figure 1A**). These results strongly suggest that the OTUs 4053 and 5722 (i.e., the strains HDS01 and HDS22, respectively) were directly involved in chemolithotrophic denitrification-dependent sulfur oxidation in the marine sediments. High-resolution microbial analysis further indicated that, aside from these two isolates, the order Chromatiales proliferated and became metabolically active (**Figure 4** and Supplementary Figure S5). This phylogenetic group may have played a complementary role in the geochemical cycles of sulfur and nitrogen in the marine sediments. On the other hand, the filamentous sulfur-oxidizing Desulfobulbaceae bacteria, which originally dominated the sediment microbial communities, did not increase under nitrate amendment, indicating that they played only minor roles in the redox process.

Notably, anaerobic microorganisms in addition to the chemolithotrophic sulfur oxidizers were metabolically activated at the end of the nitrate-amended incubation (**Table 1**). Sulfate, iron(III), and CO_2_ were originally present in the marine sediments (**Figures 1A,C** and Supplementary Figure S1E) and thus were available as electron acceptors for anaerobic respiration processes. Fine-scale analyses of Illumina sequence data successfully detected the increases in 16S rRNA transcripts from fermentative and nitrate-reducing bacteria (i.e., *Cellulomonas* sp.), iron(III) reducers (e.g., *Geobacter* spp., *Pelobacter* sp., and *Geothrix* sp.), and aceticlastic methanogens (i.e., *Methanosaeta* sp.) at day 5 (**Table 1**), although no changes in geochemical parameters such as the concentrations of TOC, iron(II), and CH_4_ were found (Supplementary Figures S1B,F). It is tempting to speculate that the carbon fixed by the chemolithotrophic sulfur oxidizers might be utilized as an electron donor by these anaerobically respiring microorganisms. In order to clarify the trophic relationships of anaerobic microorganisms in the marine sediments, isotope-tracing assays such as ultra-high-sensitivity stable isotope probing (Aoyagi et al., 2015) should be applied.

It is also noteworthy that two different types of chemolithotrophic sulfur-oxidizing bacteria (i.e., the genus *Sulfurimonas* and the order Chromatiales) coexisted in our experiments (**Figure 4**). Niche differentiation of these physiologically similar microorganisms developed during the incubation, and was a potential driving force in shaping the sediment microbial communities. A single species [the OTU 4053 (strain HDS01)] of the genus *Sulfurimonas* increased exclusively (**Figures 4A,B**), while a wide variety of species [e.g., OTUs 5722 (strain HDS22), 3944, and 1143] within the order Chromatiales became metabolically active. Almost all of the top Chromatiales bacteria in terms of 16S rRNA transcript expression levels, with the exception of the OTUs 5722 and 6560 (Supplementary Figure S5D), accounted for quite low percentages of the total microbial populations at the gene level (Supplementary Figure S5C), suggesting that these minor Chromatiales members actively dissimilated sulfur and nitrate but only marginally assimilated carbon for their growth in the sediments. Recently, genomics-based studies have reported that bacteria belonging to the genus *Sulfurimonas* and those belonging to the order Chromatiales employed different metabolic pathways for sulfur oxidation, nitrate reduction, and carbon fixation (Sievert et al., 2008; Kovaleva et al., 2011; Grote et al., 2012; Handley et al., 2014; Thomas et al., 2014). Further, some Chromatiales bacteria have been reported to be capable of growing specifically at low concentrations (i.e., 1–2 mM) of nitrate, and thereby adapting to energy-limiting conditions (Sorokin et al., 2006). These metabolic types and capacities might have triggered the distinct responses of these two sulfur oxidizers to nitrate amendment. More detailed physiological characterization and investigation into the competitive mechanism of these two isolates (the strains HDS01 and HDS22) may lead to a better understanding of the niche differentiation of sulfur-oxidizing bacteria in marine sediments.

## Conclusion

The results of this study demonstrated that the novel microorganisms *Sulfurimonas* sp. strain HDS01 and *Thioalkalispira* sp. strain HDS22 were directly involved in the chemolithotrophic denitrification-dependent sulfur oxidation in nitrate-amended marine sediments. In addition to these two sulfur-oxidizers, a variety of the Chromatiales bacteria, including minor but metabolically active microorganisms, might also play important roles in the geochemical cycles of sulfur, nitrogen, and carbon. High-resolution analyses of 16S rRNA transcripts implicated trophic interaction and niche differentiation of key microorganisms as the ecological principles underlying the reorganization of microbial communities. The two different types of sulfur oxidizers stably co-existed in the sediments and complementarily fixed carbon, leading to the metabolic activation of fermentative bacteria, ferric iron reducers, and aceticlastic methanogens. The mechanisms underlying the community reorganization should be studied to gain deeper insight into the stability and flexibility of microbial ecosystems.

## Conflict of Interest Statement

The authors declare that the research was conducted in the absence of any commercial or financial relationships that could be construed as a potential conflict of interest.

## Footnotes

1 http://metagenomics.anl.gov/

2 http://www.ddbj.nig.ac.jp
