# Supplementary material for: Dynamic transition of chemolithotrophic sulfur-oxidizing bacteria in response to amendment with nitrate in deposited marine sediments
Source: Front Microbiol. 2015 May 18;6:426. doi: 10.3389/fmicb.2015.00426 (PMC4435421; doi:10.3389/fmicb.2015.00426)

Supplementary Material

Dynamic transition of chemolithotrophic sulfur-oxidizing bacteria in response to amendment with nitrate in deposited marine sediments

Tomo Aoyagi[1†], Makoto Kimura[1†], Namiha Yamada[1], Ronald R. Navarro[1], Hideomi Itoh[2], Atsushi Ogata[1], Akiyoshi Sakoda[3], Yoko Katayama[4], Mitsuru Takasaki[5], and Tomoyuki Hori[1‡]

[1] *Environmental Management Research Institute, National Institute of Advanced Industrial Science and Technology (AIST), Tsukuba, Japan*
[2] *Bioproduction Research Institute, National Institute of Advanced Industrial Science and Technology (AIST), Sapporo, Japan*
[3] *Institute of Industrial Science, The University of Tokyo, Tokyo, Japan*
[4] *Graduate School of Agriculture, Tokyo University of Agriculture and Technology, Tokyo, Japan*
[5] *Departments of Food and Environmental Sciences, Faculty of Science and Engineering, Ishinomaki Senshu University, Ishinomaki, Japan*

†T. Aoyagi and M. Kimura contributed equally to this work

‡Correspondence: Tomoyuki Hori, Environmental Management Research Institute, National Institute of Advanced Industrial Science and Technology (AIST), Onogawa 16-1, Tsukuba, Ibaraki 305-8569, Japan.
Phone: +81 29 849 1107; Fax: +81 29 861 8326; e-mail: hori-tomo@aist.go.jp

Supplementary Figures and Tables

Table S1| Illumina sequencing data of 16S rRNA genes and transcripts, and the calculated α-diversity indices.

Figure S1| Changes in geochemical parameters during anoxic incubation of nitrate-amended marine sediments (●) and the control (○). The panels show the concentrations of ammonium **(A)**, silica **(B)**, phosphate **(C)**, TOC **(D)**, total iron **(E)**, and ferrous iron **(F)**. The error bars indicate the standard deviations of three replications.

Figure S2| Content ratios of total nitrogen (A), carbon (B), and hydrogen (C) in the solid-phase sediments during anoxic incubation of nitrate-amended marine sediments (black bar) and the control (white bar). The error bars indicate the standard deviations of three replications.

Figure S3| Representative TG/DTA patterns of the marine sediments at day 0 (A), at day 5 in the control (B), and at day 5 in the nitrate treatment (C). The black lines indicate the TG curves, and gray lines indicate DTA curves.

Figure S4| Principal coordinates analysis (PCoA) plot of 16S rRNA genes and transcripts of

Illumina sequencing data. A PCoA plot was generated from the weighted UniFrac analysis of equal numbers of sequences (*n*= 9,336). Closed and open symbols represent the nitrate-amended and non-amended control, respectively. The symbols indicate the data at the gene (triangle) and transcript levels (circle). The colors indicate the incubation days: day 0, blue; day 2, green; and day 5, red. Arrows indicate the successive time points (days).

Figure S5| The most expressed OTUs of the classes Epsilonproteobacteria (B) and Gammaproteobacteria (D) after the 5-day anoxic incubation of nitrate-amended marine sediments (black bar) and the control (white bar). OTUs are listed in descending order of relative abundance in the TG5 library **(B, D)**, and the relative abundances in the NT5 library are also shown **(A, C)**. The white bars indicate their relative abundances in the CG5 and CT5 libraries.

Table S1 Illumina sequencing data of 16S rRNA genes and transcripts, and the calculated α-diversity indices

16S rRNA	Sequence library name	No. of sequences	Alpha-diversity[a]		
			Chao1	Shannon	1/Simpson
Genes	G0	40,869	2620±159	9.27±0.34	130.1±6.4
	NG2	23,005	4593±184	8.89±0.03	84.7±1.6
	NG5	40,169	3131±294	5.50±0.05	5.4±0.1
	CG2	34,022	4951±157	9.37±0.03	137.7±4.9
	CG5	33,723	4730±189	9.24±0.02	110.2±5.4
Transcripts	T0	9,336	4313±249	6.81±0.02	16.1±0.2
	NT2	13,527	4237±198	6.71±0.03	18.8±0.5
	NT5	16,191	3020±219	5.70±0.04	15.8±0.3
	CT2	36,721	4594±213	6.89±0.04	18.0±0.5
	CT5	33,088	3950±207	6.78±0.04	17.8±0.3

[a] Each diversity index was calculated based on an equivalent number of sequences (n= 7,374) subsampled 10 times from the original libraries. The average data and the standard deviations of 10-time subsamples are shown.

54

Aoyagi *et al.*, Figure S1

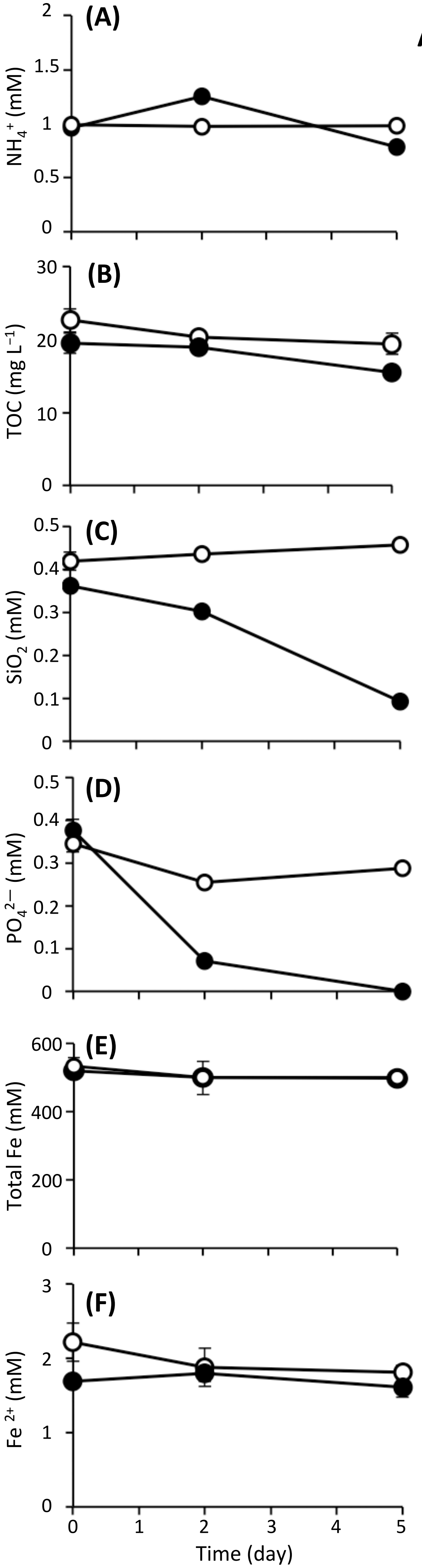

Aoyagi *et al.*, Figure S2

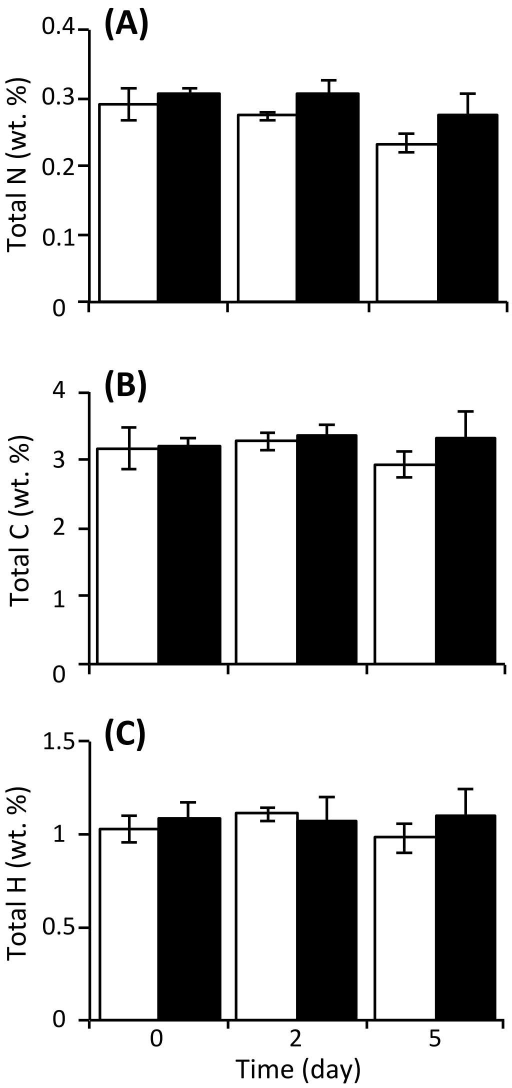

Aoyagi *et al.*, Figure S3

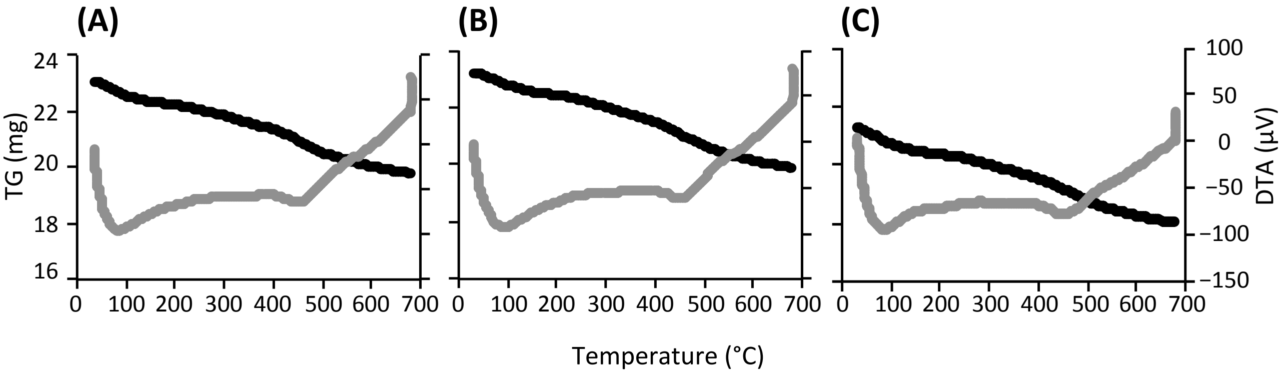

Aoyagi *et al.*, Figure S4

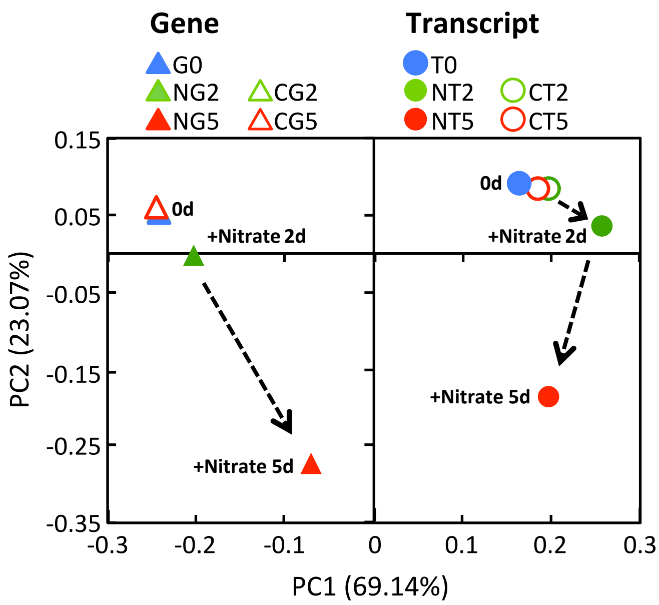

Aoyagi *et al.*, Figure S5

Epsilonproteobacteria

OTU	Closely related species	Similarity (%)	Acc. No.
4053	*Sulfurimonas denitrificans*	98.4	NR074133
2169	*Sulfurimonas gotlandica*	95.6	NR121690
5598	*Sulfurimonas gotlandica*	92.0	NR121690
12272	*Sulfurimonas autotrophica*	90.1	NR074451
16768	*Sulfurimonas gotlandica*	96.4	NR074133
10381	*Sulfurimonas denitrificans*	96.8	NR074133
9364	*Sulfurimonas autotrophica*	91.2	NR074451
9806	*Sulfurimonas paralvinellae*	90.4	NR041439
8028	*Sulfurimonas denitrificans*	96.8	NR074133
555	*Sulfurimonas denitrificans*	95.2	NR_074133

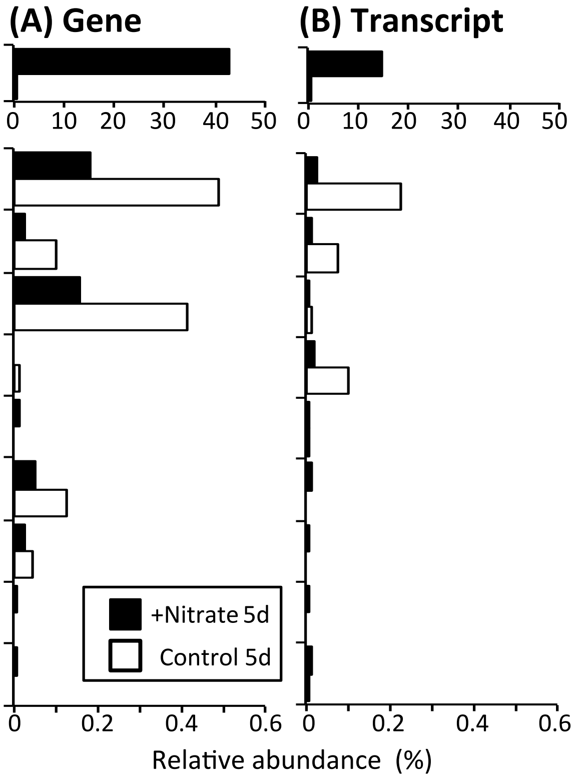

Gammaproteobacteria

OTU	Closely related species	Similarity (%)	Acc. No.
3944	*Thioalbus denitrificans*	100	NR122087
5722	*Thioalkalispira microaerophila*	96.8	NR025239
1143	*Thioprofundum hispidum*	96.0	NR112620
13536	*Thioalkalispira microaerophila*	97.6	NR025239
11183	*Thioalbus denitrificans*	96.8	NR122087
12681	*Thioprofundum lithotrophicum*	96.4	NR112829
15348	*Thioprofundum lithotrophicum*	95.3	NR112829
6560	Thiotrichales bacterium	92.5	HQ675680
14834	*Thioalbus denitrificans*	96.8	NR122087
12930	*Thioalkalispira microaerophila*	97.6	NR025239

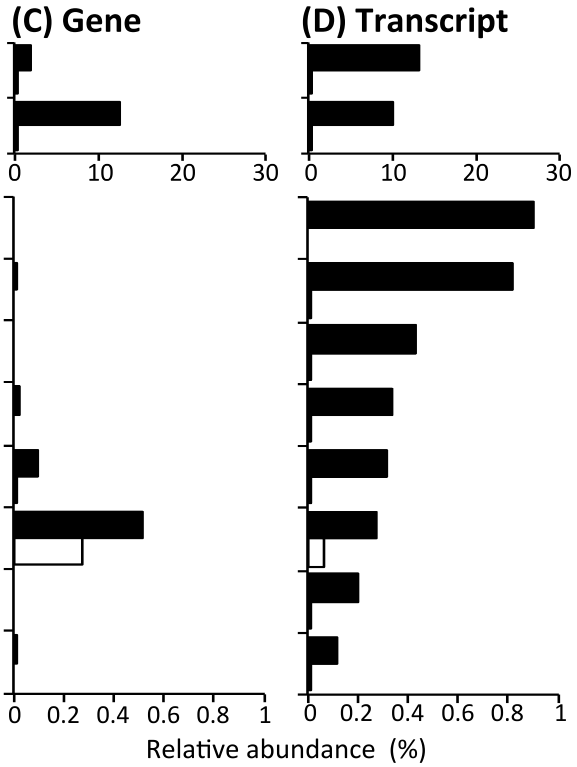

Supplement: Supplementary file 1 [file Data_Sheet_1.PDF]
